# Graduates of the Veterinary Department of Harvard University

**Published:** 1887-07

**Authors:** 


### GRADUATES OF THE VETERINARY DEPARTMENT OF HARVARD UNIVERSITY.

At the commencement exercises of Harvard University, held at Cambridge, Mass., on June 29, 1887, the following gentlemen received the degree of *Medicinæ Doctor Veterinariæ*:

Francis Boyd Carleton, Salem, Massachusetts,
Edwin James Castle, Mithuen, Massachusetts,
Daniel Emerson (Ph. G.), Boston, Massachusetts,
William Alvan Hitchcock, Madden, Massachusetts,
Emmet Walter Roche, West Roxbury, Mass.,
Ernest Charles Schroder, Baltimore, Maryland,
Harrison Whitney, (A.B.), Harrison, Maine.

